# Supplementary material for: A Markerless Gene Deletion System in Streptococcus suis by Using the Copper-Inducible Vibrio parahaemolyticus YoeB Toxin as a Counterselectable Marker
Source: Microorganisms. 2021 May 19;9(5):1095. doi: 10.3390/microorganisms9051095 (PMC8160710; doi:10.3390/microorganisms9051095)
Supplement: Supplementary file 1 [file microorganisms-09-01095-s001.zip › microorganisms-1191886-supplementary.pdf]

Table S1. Primers used in this study.

Primer	Sequence (5'-3') [1]	Size (bp)	Target gene
P*copA*-F	CG**GGATCC**TTTGGGGAACCTTTGGAG	246	The promoter of *S. suis copA*
P*copA*-R	TACTCATCAAAGGTTACTCCTTTTCCGACTAC		
yoeB$_{Vp}$-F	GGAGTAACCTTTGATGAGTAGTAGTCAACGTTTATT	427	The *yoeB*$_{Vp}$ gene of *V. parahaemolyticus*
yoeB$_{Vp}$-R	CG**GAATTC**TGTAAAATATAACGCCCAATTAA		
P*copA*-R2	GGATTCCCATAGGTTACTCCTTTTCCGACTAC	246	Combine with P*copA*-F to amplify P*copA*
yoeB$_{Ss}$-F	GGAGTAACCTATGGGAATCCATTTTACAGAC	258	The *yoeB*$_{Ss}$ gene of *S. suis*
yoeB$_{Ss}$-R	CGGAATTCTCACCTATAATGATCCTTTAGCG		
Q*yoeB*$_{Vp}$-F	CGACGAGACCAAAAACCAGATAAG	158	An internal region of *yoeB*$_{Vp}$
Q*yoeB*$_{Vp}$-R	GATGATGCTTGGGATGACTACCTG		
Q*pmtA*-F	CGATGTTGTCCGCAATGTCAC	118	An internal region of *pmtA*
Q*pmtA*-R	AACCATCGCTTCTCCTTGTGC		
Q16S-F	TAGTCCACGCCGTAAACGATG	159	An internal region of 16S rRNA
Q16S-R	TAAACCACATGCTCCACCGC		
spc-F	GTTCGTGAATACATGTTATAATAACT	1130	The spectinomycin gene
spc-R	GTTCCCCAAAGTTTTCTAAAATCTGATTACCAAT		
P*copA*-*yoeB*$_{Vp}$-F	TTTAGAAAACTTTGGGGAACCTTTGGA	673	*yoeB*$_{Vp}$ and the promoter P*copA*
P*copA*-*yoeB*$_{Vp}$-R	TGTAAAATATAACGCCCAATT		
pmtA-LA-F	TGTGTAGCCCTCATTTATTGG	1166	Left arm of *pmtA*
pmtA-Fir-LA-R	TATTCACGAACCTATCAACCTCCCACTCGG		
pmtA-SCIY-F	GAGGTTGATAGGTTCGTGAATACATGTTATAATAACT	1803	The SCIY cassette
pmtA-SCIY-R	ACGACCGCTCTTGTAAAATATAACGCCCAATT		
pmtA-Fir-RA-F	TATATTTTACAAGAGCGGTCGTGATGATGT	1170	Right arm of *pmtA*
pmtA-RA-R	ATAGGTCTTGCTGTGCTTGG		
pmtA-Sec-LA-R	CGACCGCTCTCTATCAACCTCCCACTCGG		
pmtA-Sec-RA-F	AGGTTGATAGAGAGCGGTCGTGATGATGT		
pmtA-in-F	GTCTTTTCTTGGTCACCCG	755	An internal region of *pmtA*
pmtA-in-R	TCAAAAACAGCGACTTTCATT		
pmtA-out-F	CCAGAGATTTTCAATAAGGCA	4199/2472	A fragment containing *pmtA*

pmtA-out-R	CATAGGGATAGAAGGTCGCA		
perR-LA-F	GATGTTGTCCGCAATGTCAC	1135	Left arm of *perR*
perR-Fir-LA-R	ATTCACGAACCATCTACGGCATTTGTCCTG		
perR-SCIY-F	GCCGTAGATGGTTCGTGAATACATGTTATAATAACT	1803	The SCIY cassette
perR-SCIY-R	ATCTAAAAGAATGTAAAATATAACGCCCAATT		
perR-Fir-RA-F	TATATTTTACATTCTTTTAGATGTTGGATGACGT	1138	Right arm of *perR*
perR-RA-R	GTCGGAATGACCTCAAAACC		
perR-Sec-LA-R	ATCTAAAAGAACATCTACGGCATTTGTCCTG		
perR-Sec-RA-F	GCCGTAGATGTTCTTTTAGATGTTGGATGACGT		
perR-in-F	TCAAGCTCGGGATTTCTACA	241	An internal region of *perR*
perR-in-R	GATGACCACCCTAGTGCTGA		
perR-out-F	TCATCGCCAAGATAGGTCG	2794/2444	A fragment containing *perR*
perR-out-R	AGAGGGAAGCAAGCCAATC		
lysR-LA-F	ATACCTGCTGGTGTCTCGG	1250	Left arm of *lysR*
lysR-Fir-LA-R	ATTCACGAACACTGCCTGTTTCTGCAATTT		
lysR-SCIY-F	AACAGGCAGTGTTCGTGAATACATGTTATAATAACT	1803	The SCIY cassette
lysR-SCIY-R	GCCTTTTCGTTGTAAAATATAACGCCCAATT		
lysR-Fir-RA-F	TATATTTTACAACGAAAAGGCATTGCTGTC	1264	Right arm of *lysR*
lysR-RA-R	CAACACCTTCGCCGATAAT		
lysR-Sec-LA-R	GCCTTTTCGTACTGCCTGTTTCTGCAATTT		
lysR-Sec-RA-F	AACAGGCAGTACGAAAAGGCATTGCTGTC		
lysR-in-F	CGCCAACTATTTAGCGTCTC	329	An internal region of *lysR*
lysR-in-R	GGGAAATCTACCAAATCATCG		
lysR-out-F	TTCGGATAAGACCGCCTC	3460/2679	A fragment containing *lysR*
lysR-out-R	CCTTTCTTACTTGGTCACATTCT		

[1] The bold sequences are restriction sites.

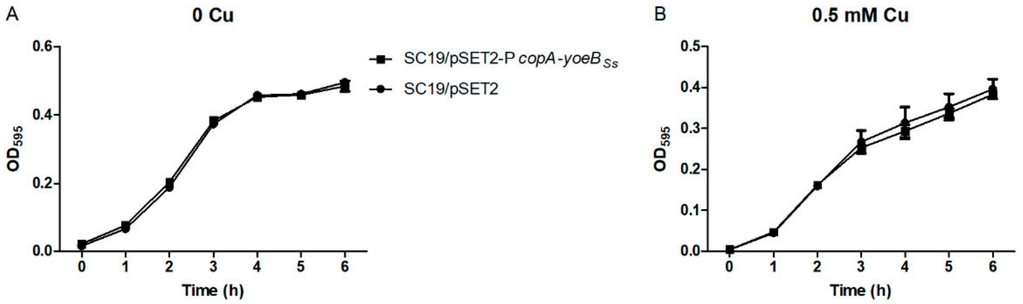

Figure S1. YoeB$_{Ss}$ expression had no significant effect on *S. suis* growth in liquid media. The SC19/pSET2-P*copA*-*yoeB*$_{Ss}$ and SC19/pSET2 strains were grown in the absence (A) and presence of 0.5 mM $CuSO_4$ (B). At least three independent experiments were performed; the data shown are the means ± SDs from three wells in a representative experiment.

Text S1. DNA sequence of the SCIY cassette (5'-3').

“GTTCGTGAATACATGTTATAATAACTATAACTAATAACGTAACGTGACTGGCAAGAG
ATATTTTTAAAACAATGAATAGGTTTACACTTACTTTAGTTTTATGGAAATGAAAGAT
CATATCATATATAATCTAGAATAAAATTAACTAAAATAATTATTATCTAGATAAAAA
ATTTAGAAGCCAATGAAATCTATAAATAAACTAAATTAAGTTTATTTAATTAACAACT
ATGGATATAAAATAGGTACTAATCAAAATAGTGAGGAGGATATATTTGAATACATAC
GAACAAATTAATAAAGTGAAAAAAATACTTCGGAAACATTTAAAAAATAACCTTATT
GGTACTTACATGTTTGGATCAGGAGTTGAGAGTGGACTAAAACCAAATAGTGATCTT
GACTTTTTAGTCGTCGTATCTGAACCATTGACAGATCAAAGTAAAGAAATACTTATAC
AAAAAATTAGACCTATTTCAAAAAAAATAGGAGATAAAAGCAACTTACGATATATTG
AATTAACAATTATTATTCAGCAAGAAATGGTACCGTGGAATCATCCTCCCAAACAAG
AATTTATTTATGGAGAATGGTTACAAGAGCTTTATGAACAAGGATACATTCCTCAGAA
GGAATTAAATTCAGATTTAACCATAATGCTTTACCAAGCAAAACGAAAAAATAAAA
GAATATACGGAAATTATGACTTAGAGGAATTACTACCTGATATTCCATTTTCTGATGT
GAGAAGAGCCATTATGGATTCGTCAGAGGAATTAATAGATAATTATCAGGATGATGA
AACCAACTCTATATTAACTTTATGCCGTATGATTTTAACTATGGACACGGGTAAAATC
ATACCAAAAGATATTGCGGGAAATGCAGTGGCTGAATCTTCTCCATTAGAACATAGG
GAGAGAATTTTGTTAGCAGTTCGTAGTTATCTTGGAGAGAATATTGAATGGACTAATG
AAAATGTAAATTTAACTATAAACTATTTAAATAACAGATTAAAAAAATTATAAAAAA
ATTGAAAAAATGGTGGAAACACTTTTTTCAATTTTTTTGTTTTATTATTTAATATTTGGG
AAATATTCATTCTAATTGGTAATCAGATTTTAGAAAACTTTGGGGAACCTTTGGAGGT
TGGAGATAGAGCGAACCTAGTTCGTATCGATATGATGTAGATAGAACACTGTTCCTTT
TATATTTCAAGGGAACAGGCTGAAAACCTCCACAGGAGCTTTTCAGTCATCAAATCA
AGTCAACAACGTCTGATTTTGATTTTCGAAGAGTATTAATCAAAAATGTTTACAAACG
TAGTCGTATATTGTATAATTTGTTTACAGATGTAGTCGGAAAAGGAGTAACCTTTGAT
GAGTAGTAGTCAACGTTTATTATCGTGGACTGATGATGCTTGGGATGACTACCTGTAT
TGGCAAACTCAAGACAAGAAAACACTCAAGCGCATCAATAAACTCATCAATGATGTT
AAGCGCTCTCCATTTGAGGGCATTGGTAAACCAGAGCCGTTAAAAGAGAACTTATCT
GGTTTTTGGTCTCGTCGTATTGATGATACTAATAGGCTTGTTTACGCAGTCGATGATCA
AGCGATAACGATAATTTCATGTCGTTACCACTACTAAATCAGGTTCAGCGATCTGACA
TCTAACAAACAATTTAAGAGTGATTCTCAACGCTTGGCATTTTTCATTCCATCGTTGGG
TTTTGTGTTTAAGGTGTAATGGTTTCGTTTCGTGGTAGCGTTGCTCACACCTTAATTGG
GCGTTATATTTTACA”
